# Supplementary material for: Perceived Barriers and Intentions to Receive COVID-19 Vaccines: Psychological Distress as a Moderator
Source: Vaccines (Basel). 2023 Jan 28;11(2):289. doi: 10.3390/vaccines11020289 (PMC9967752; doi:10.3390/vaccines11020289)
Supplement: Supplementary file 1 [file vaccines-11-00289-s001.zip › vaccines-2155843-supplementary.pdf]

Questionnaires

Dear participant,

We are a team of researchers at National Economics University, Vietnam. We interested in the study of intention to receive a COVID-19 vaccine among Vietnamese citizens. To this end, we are carrying out this anonymous survey in different regions (North, Central, and South) of Vietnam. We are hoping you will help us out by answering the questions below. Please try to answer each question even if you feel it is repetitive. Your benefits in participating are that you will help to gain new knowledge on intention or hesitancy to receive Covid-19 vaccine in Vietnam that will be disseminated in final report, papers. We would like to assure you that your responses are completely anonymous and confidential and that you are under no obligation to participate and can redraw at any stage. There is no right or wrong answer; we just need an honest answer, one that represents your true perception and experience.

Please answer all questions and thank you for your time.

SECTION 1. DEMOGRAPHIC INFORMATION

1. Gender: □1 Male □2 Female
2. Age: □1 18-28 years old
   □2 29-38 years old
   □3 39-48 years old
   □4 49-58 years old
   □5 Over 59 years old
3. Monthly income:
   □1 Less than 10 million VND
   □2 From 10 to 20 million VND
   □3 From 20 to 30 million VND
   □4 Over 30 million VND
4. Educational level:

□1 High school
□2 Bachelor's degree
□3 Master/PhD degree

5. Material status
□1 Single
□2 Married

6. Did you receive a COVID-19 vaccine?
□1 Yes
□2 Not yet

7. What kind of COVID-19 vaccine would you like to receive?
□1 AustraZeneca □3 Moderna □5 Sinopharm □7 Nanocovax
□2 Pfizer □4 Sputnik-V □6 Johnson & Johnson □8 Others.

SECTION 2. COVID-19 VACCINE

Please answer the following questions with seven-Likert scale:

①	②	③	④	⑤	⑥	⑦
Strongly disagree	*Disagree*	*Somewhat disagree*	*Neutral*	*Somewhat agree*	*Agree*	*Somewhat agree*

**A. Intention to receive a COVID-19 vaccine**							
I try to get COVID-19 vaccines	①	②	③	④	⑤	⑥	⑦

I actually get vaccinated for COVID-19	①	②	③	④	⑤	⑥	⑦
I get vaccinated if a physician offered me COVID-19 vaccines	①	②	③	④	⑤	⑥	⑦
I am ready to do anything to receive a COVID vaccine	①	②	③	④	⑤	⑥	⑦
My goal is to receive a COVID-19 as soon as possible	①	②	③	④	⑤	⑥	⑦
I will make every effort to receive a COVID vaccine	①	②	③	④	⑤	⑥	⑦
I am a very seriously thought of receiving a COVID vaccine	①	②	③	④	⑤	⑥	⑦
I have the firm intention to receive a COVID vaccine	①	②	③	④	⑤	⑥	⑦
**B. Attitude towards receiving a COVID-19 vaccine**							
Receiving a COVID-19 vaccine implies more advantages than disadvantages to me	①	②	③	④	⑤	⑥	⑦
Receiving a COVID-19 vaccine is attractive for me	①	②	③	④	⑤	⑥	⑦
If I had the opportunity, I would like to receive a COVID-19 vaccine	①	②	③	④	⑤	⑥	⑦
Receiving a COVID-19 vaccine would entail great satisfactions for me	①	②	③	④	⑤	⑥	⑦
Among various options, I would rather receive a COVID-19 vaccine	①	②	③	④	⑤	⑥	⑦
**C. Self-efficacy**	①	②	③	④	⑤	⑥	⑦
I will be able to get the vaccines to prevent contracting COVID-19	①	②	③	④	⑤	⑥	⑦
I will be easy for me to get the vaccines to protect myself from COVID-19	①	②	③	④	⑤	⑥	⑦
Getting vaccinated to prevent COVID-19 is convenient	①	②	③	④	⑤	⑥	⑦
I will be able to get the vaccines to prevent contracting COVID-19	①	②	③	④	⑤	⑥	⑦
I will be easy for me to get the vaccines to protect myself from COVID-19	①	②	③	④	⑤	⑥	⑦
**D. Perceived barriers**	①	②	③	④	⑤	⑥	⑦
I will have side effects from the COVID-19 vaccine	①	②	③	④	⑤	⑥	⑦
The COVID-19 vaccine will be safe	①	②	③	④	⑤	⑥	⑦
I will get sick from the COVID-19 vaccine	①	②	③	④	⑤	⑥	⑦
I will die from the COVID-19 vaccine	①	②	③	④	⑤	⑥	⑦
The COVID-19 vaccine will be painful	①	②	③	④	⑤	⑥	⑦
It will be hard for me to get the COVID-19 vaccine	①	②	③	④	⑤	⑥	⑦
There will not be enough of the COVID-19 vaccine for me	①	②	③	④	⑤	⑥	⑦
The COVID-19 vaccine will cost me a lot of my own money	①	②	③	④	⑤	⑥	⑦
